# Chronic intermittent psychological stress promotes macrophage reverse cholesterol transport by impairing bile acid absorption in mice

**DOI:** 10.14814/phy2.12402

**Published:** 2015-05-11

**Authors:** Reija Silvennoinen, Helena Quesada, Ilona Kareinen, Josep Julve, Leena Kaipiainen, Helena Gylling, Francisco Blanco-Vaca, Joan Carles Escola-Gil, Petri T Kovanen, Miriam Lee-Rueckert

**Affiliations:** 1 Wihuri Research InstituteHelsinki, Finland; 2 IIB Sant Pau, Departament de Bioquímica i Biologia Molecular, Universitat Autònoma de Barcelona-CIBER de Diabetes y Enfermedades Metabolicas AsociadasBarcelona, Spain; 3 Internal Medicine, University of Helsinki and Helsinki University HospitalHelsinki, Finland

**Keywords:** Bile acids, physical restraint, psychological stress, reverse cholesterol transport

## Abstract

Psychological stress is a risk factor for atherosclerosis, yet the pathophysiological mechanisms involved remain elusive. The transfer of cholesterol from macrophage foam cells to liver and feces (the macrophage-specific reverse cholesterol transport, m-RCT) is an important antiatherogenic pathway. Because exposure of mice to physical restraint, a model of psychological stress, increases serum levels of corticosterone, and as bile acid homeostasis is disrupted in glucocorticoid-treated animals, we investigated if chronic intermittent restraint stress would modify m-RCT by altering the enterohepatic circulation of bile acids. C57Bl/6J mice exposed to intermittent stress for 5 days exhibited increased transit through the large intestine and enhanced fecal bile acid excretion. Of the transcription factors and transporters that regulate bile acid homeostasis, the mRNA expression levels of the hepatic farnesoid X receptor (FXR), the bile salt export pump (BSEP), and the intestinal fibroblast growth factor 15 (FGF15) were reduced, whereas those of the ileal apical sodium-dependent bile acid transporter (ASBT), responsible for active bile acid absorption, remained unchanged. Neither did the hepatic expression of cholesterol 7*α*-hydroxylase (CYP7A1), the key enzyme regulating bile acid synthesis, change in the stressed mice. Evaluation of the functionality of the m-RCT pathway revealed increased fecal excretion of bile acids that had been synthesized from macrophage-derived cholesterol. Overall, our study reveals that chronic intermittent stress in mice accelerates m-RCT specifically by increasing fecal excretion of bile acids. This novel mechanism of m-RCT induction could have antiatherogenic potential under conditions of chronic stress.

## Introduction

Impaired regulation of systemic cholesterol and bile acid homeostasis is involved in the pathophysiology of several common disorders such as hypercholesterolemia and cardiovascular disease (Rajaratnam et al. 2001; Matthan et al. 2009), gallstone disease (Portincasa et al. 2006), and type 2 diabetes (Haeusler et al. 2013). Over the last decades, epidemiological studies have evidenced the exacerbating or even founding effect of psychological stress, and particularly of occupational stress, on the risk of coronary artery disease (Bosma et al. 1997; Bjorntorp 1999; Kivimaki et al. 2012; Nyberg et al. 2013). The secretion of adrenal glucocorticoids is a classic endocrine response to stress. Endogenous hypercortisolism of Cushing's syndrome and the use of high-dose glucocorticoid therapy also associate with cardiovascular mortality and atherosclerosis (Sugihara et al. 1992; Del Rincon et al. 2003; Neary et al. 2013). Despite these rather uniform notions about the harmful effects of various forms of stress on cardiometabolic health, animal studies on the effects of psychological stress and glucocorticoid treatment on atherosclerosis exhibit varying results (Kaplan et al. 1983; McCabe et al. 2002; Bernberg et al. 2009, 2012), and the underlying mechanisms connecting stress to the etiology of atherosclerotic cardiovascular disease remain elusive.

Macrophage-specific RCT (m-RCT) is considered one of the major cardioprotective mechanisms mediated by HDL (Cuchel and Rader 2006). The multistep process is initiated in the arterial intima where HDL particles accept cholesterol from macrophage foam cells via ATP-binding cassette transporters A1 and G1 (ABCA1, ABCG1). Circulating HDL deliver their cholesterol cargo into the liver where a fraction of the cholesterol is converted into bile acids (Wang et al. 2004; Alexander et al. 2011). From the liver, cholesterol, bile acids, and phospholipids are secreted into the intestine as constituents of the bile. Eventually, efficiency of intestinal reabsorption determines the fecal excretion rate of both cholesterol and bile acids (Sehayek and Hazen 2008). The efficiency of cholesterol absorption from the intestine is extremely variable, whereas that of bile acids is uniformly very high*,* 90–95% of intestinal bile acids being delivered back into the liver (Vuoristo and Miettinen 2000). Complex regulatory circuits governed by the intestinal and hepatic farnesoid X receptor (FXR) promote the efficient enterohepatic circulation of bile acids and ensure that their levels in the intestine remain optimal for endothelial integrity, lipid absorption, and various signaling functions (Hylemon et al. 2009; Kemper 2011). Metabolic and hormonal cues such as glucocorticoids are capable of modulating the FXR-mediated bile acid homeostasis on both the organ and whole-body level (Lu et al. 2012; Rosales et al. 2013; Out et al. 2014).

Several lines of evidence establish that the rate of m-RCT is susceptible to modulation in the gut (Lee-Rueckert et al. 2013). Previously, we demonstrated that exposure of C57Bl/6J mice to a single episode of physical restraint stress increases the level of serum corticosterone and the rate of m-RCT (Silvennoinen et al. 2012). The stimulation of m-RCT resulted from reduced intestinal absorption of cholesterol which could be explained by peroxisome proliferator activator *α* (PPAR*α*)-dependent downregulation of the Niemann-Pick-type C1-like 1 (NPC1L1), a transmembrane protein critical for the uptake of cholesterol across the plasma membrane of the intestinal enterocyte. In addition to the single episode of stress, repeated episodes of stress, when applied once or twice per day for up to five consecutive days, increased the m-RCT rate in the stressed mice. However, whether the acute and repeated stress models shared a common stimulatory mechanism on m-RCT remained elusive. Importantly, the two stress models differ fundamentally because multiple elevations in glucocorticoid secretion due to repeated episodes of stress challenge the quality and quantity of an acute adaptive stress reaction (Herman 2013). Furthermore, habituation, a process that gradually dampens the glucocorticoid response to a familiar stressor, may limit the impacts of chronic stress. The results obtained in this study indicate that chronic intermittent psychological stress, in sharp contrast to acute psychological stress (Silvennoinen et al. 2012), does not affect intestinal cholesterol absorption, but instead, transiently disrupts the intestinal absorption of bile acids and consequently stimulates the antiatherogenic m-RCT pathway.

## Materials and Methods

### Mice

9–13-week-old female C57BL/6JOlaHsd mice from Harlan Laboratories (Venray, the Netherlands) were housed 3–5 per cage under controlled conditions for the light/dark cycle, temperature, and humidity. The animals were kept in the same animal facility for at least 1 week before the experiments. Mice were fed a standard chow diet (2016 Teklad Global, Harlan Laboratories), and food and water were provided ad libitum. The experiments were conducted in conformity with Finnish and Spanish regulations, and the protocols were approved by the Finnish National Animal Experiment Board in Finland and by the Institutional Animal Care Committee of the Institut de Recerca de l'Hospital de la Santa Creu i Sant Pau, Barcelona, in Spain.

### Chronic intermittent psychological stress

**Figure 1:**
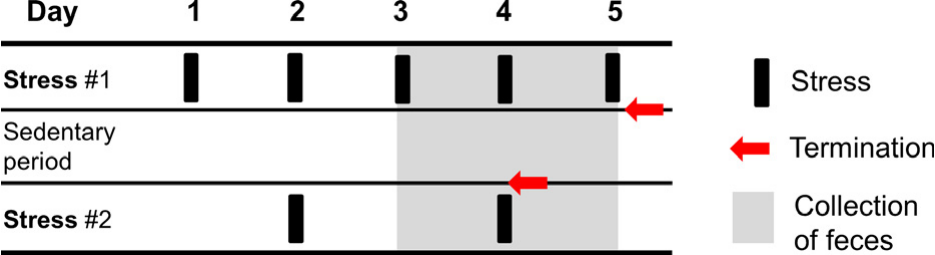
Schematic illustration of the chronic intermittent stress regime. The 5-day stress regime included a total of 14 h of restraint stress divided in 2-h episodes separated by sedentary periods. The regime was terminated on day 4 (after a 4-h sedentary period) or on day 5 (immediately after stress). Feces were collected for 24 or 48 h, depending on the assay. Mice were euthanized immediately after the final stress episode.

**Figure 2:**
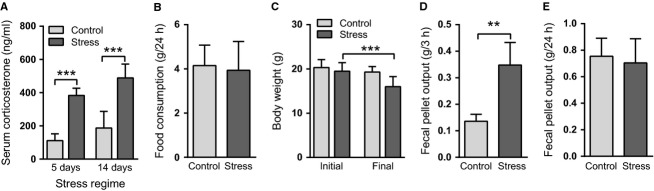
Chronic intermittent stress modulates serum corticosterone, body weight, and fecal pellet output. (A) Concentration of corticosterone (ng/mL) in serum obtained from mice subjected to repeated restraint stress for five (*N* = 15 mice/group) or 14 days (*N* = 5 mice/group) and from respective control mice. Blood samples were drawn immediately after the final stress episode of the regimes. (B) Food consumption (g/mouse) during the final 24 h of the 5-day stress regime (*N* = 5 mice/group). (C) Body weight (g) at the start (initial) and at the end (final) of the 5-day stress regime (*N* = 10 mice/group). (D) Fecal pellet output during a 3-h stress episode at the end of the 5-day stress regime (*N* = 5 mice/group). (E) Fecal pellet output during the two final days of the 5-day stress regime (*N* = 10 mice/group). **P < 0.01; ***P < 0.001. All data are presented as mean + SD.

Mice were exposed to chronic intermittent psychological stress by placing them into plastic restraint cylinders (Harvard Apparatus, Cambridge, MA) for 2–3 h at room temperature (Silvennoinen et al. 2012). The restraint sessions were applied between 9 am and 6 pm to prevent interference of circadian rhythms, and welfare of the animals was monitored regularly during the immobilization. Nonstressed control mice were kept in their cages isolated from stressed mice to avoid any acoustic or olfactory communication between the groups. Both groups of mice were deprived of food and water during the time period in which the stressed group was immobilized. Mice were stressed repeatedly during 5 days according to the schedule presented in Figure1. Stressed mice were returned to their home cages during sedentary periods in between the stress episodes. To study the changes occurring during such a sedentary period within the 5-day stress regime, a group of mice was killed 4 h after the first stress episode of day 4 (Fig.1, Table1 and Fig. 7). In addition, serum corticosterone was measured in a group of mice that were exposed to an extended restraint stress regime of 14 days (Fig.2A) including five sessions of 2-h stress and 10 sessions of 1-h stress (total 20 h of restraint). Both stressed and control mice were killed by isoflurane inhalation followed by cervical dislocation at the end of the experiments.

**Table 1:** Effect of chronic intermittent stress on serum bile acids and lipids in mice

	Control	Stress	*N*	*P*-value
Immediately after stress				
Bile acids (*μ*mol/L)	4.8 (1.8)	3.1 (1.3)	10	**0.02**
Total CHOL (mmol/L)	2.6 (0.3)	2.1 (0.4)	16	**0.0004**
HDL CHOL (mmol/L)	1.4 (0.3)	1.3 (0.3)	16	0.24
Non-HDL CHOL (mmol/L)	1.1 (0.4)	0.8 (0.4)	16	**0.02**
Serum TG (mmol/L)	0.6 (0.2)	0.5 (0.2)	16	0.07
After the sedentary period				
Bile acids (*μ*mol/L)	5.3 (2.8)	4.7 (2.9)	8	0.09
Total CHOL (mmol/L)	2.2 (0.3)	2.2 (0.2)	6	0.99
HDL CHOL (mmol/L)	1.5 (0.5)	1.5 (0.5)	6	0.71
Non-HDL CHOL (mmol/L)	1.2 (0.4)	1.0 (0.8)	6	0.24
Serum TG (mmol/L)	0.5 (0.2)	0.6 (0.2)	5	0.41

Values are reported as means with standard deviations in parentheses. CHOL = cholesterol, TG = triglycerides. The data in the first five rows are from mice killed immediately after stress on day 5, and the following five rows represent data from mice euthanized 4 h after stress on day 4 (s*edentary period)*. All mice were fasted for 2 h before blood withdrawal. Statistically significant differences between control and stressed mice are bolded.

### Macrophage-to-feces RCT

For the in vivo measurement of m-RCT (Silvennoinen et al. 2012), [1,2-^3^H(N)]cholesterol (Perkin Elmer, Waltham, MA) – and acetyl-LDL-loaded J774A mouse macrophages (ATCC, Manassas, VA) were injected (1–2 × 10^6^ cells, 3–4 × 10^6^ dpm/mouse in 0.4 mL of saline) intraperitoneally under light isoflurane anesthesia. To prevent coprophagy, mice were housed in grid-bottom cages after the injection. After 4–48 h, depending on the experiment, mice were exsanguinated by cardiac puncture under terminal isoflurane anesthesia. After bleeding, the liver and feces were collected. ^3^H-radioactivity was quantified by liquid scintillation counting (LSC) in total serum and in the HDL fraction after precipitation of apolipoprotein B-containing lipoproteins with phosphotungstate-magnesium. Lipids were extracted from the liver and feces with isopropyl alcohol-hexane (2:3). After a 24-h extraction, the hexane layer was collected, evaporated, and ^3^H-radioactivity quantified by LSC. The ^3^H-radioactivity in the remaining aqueous phase in the fecal samples (containing [^3^H]bile acids) was counted separately. The amount of ^3^H-radioactivity present in each sample is expressed as percentage of the injected dose.

### Measurement of [^3^H]cholesterol efflux from cultured mouse macrophage foam cells

Cholesterol efflux promoted by mouse serum was evaluated as described previously (Silvennoinen et al. 2012). The mouse J774A macrophages were cultured and loaded with cholesterol as for the m-RCT assay. Macrophage foam cells were incubated for 3 h in Roswell Park Memorial Institute 1640 (RPMI 1640) medium containing 10 IU/mL hirudin and 2.5% (v/v) serum from control and stressed mice. After 3 h, the media and cells were collected separately and the ^3^H-radioactivity was quantified by LSC. Cholesterol efflux (%) was calculated as [dpm medium/(dpm cells + dpm medium)] × 100. Cholesterol efflux into the incubation medium in the absence of serum was considered as basal efflux, and was subtracted from the efflux values obtained in the presence of serum.

### Intestinal cholesterol absorption

The efficiency of intestinal cholesterol absorption was determined by the fecal dual isotope ratio method using [^3^H]sitostanol as a nonabsorbable recovery standard (Wang and Carey 2003). Mice received a gastric bolus of 150 *μ*L olive oil containing 1 *μ*Ci of [4-^14^C]cholesterol together with 2 *μ*Ci of [5,6-^3^H]sitostanol (both from American Radiolabeled Chemicals, St. Louis, MO). After 3 or 24 h, blood, stomach, intestines, and feces were collected, and lipids were extracted from the tissues with hexane-isopropanol. Radioactivity was quantified by LSC as in the m-RCT assay. Serum was separated from blood and measured directly by LSC. The amount of ^3^H- and ^14^C-radioactivity present in each sample is expressed as percentage of injected dose. The fractional cholesterol absorption was calculated according to the formula: ([^14^C]/[^3^H] (in dosing solution) − [^14^C]/[^3^H] (in sample))/[^14^C]/[^3^H] (in dosing solution).

### Distribution of orally administered [^3^H]taurocholate

Mice received an intragastric gavage of 5 *μ*Ci [^3^H]taurocholic acid (Perkin Elmer) dissolved in saline containing 3% ethanol (v/v) (Mendez-Gonzalez et al. 2010). After 24 h, mice were killed and blood, liver, the content of the gallbladder and small and large intestine, and feces were collected. Bile acids were extracted from the liver, small and large intestinal walls and contents, and feces with ethanol for 24 h at room temperature. After evaporation of ethanol, the extracts were redissolved in scintillation liquid and radioactivity was quantified by LSC. Serum was separated from blood and counted directly. Bile acid extraction efficiency from the collected tissues was determined by using labeled taurocholic acid as an internal standard, and it ranged from ~30% (liver) to ~80% (small intestinal contents). Less than 1% of the dose remained in the stomach. The results were corrected for the losses in bile acid extraction based on the recoveries of the internal standard, and are expressed as percentages of the gavaged dose.

### Quantitative real-time PCR

Total RNA was isolated using the trizol RNA isolation method (Gibco/BRL, Grand Island, NY) from livers, and small intestine pools made with equal contributions of the duodenal, jejunal, and ileal tissue sections (Silvennoinen et al. 2012). cDNA was generated using Oligo(dT)23 (Sigma-Aldrich, St. Louis, MO) and M-MLV Reverse Transcriptase, RNase H Minus, Point Mutant (Promega, Madison, WI) and was subjected to quantitative RT-PCR amplification using the TaqMan Master Mix (Applied Biosystems, Foster City, CA). Specific TaqMan probes (Applied Biosystems) were used for each gene: Mm00443451_m1 for *Lxrα*, Mm00440939_m1 for *Pparα*, Mm00446241_m1 for *Abcg5*, Mm00445970_m1 for *Abcg8*, Mm00450236_m1 for *Sr-b1*, Mm01184322_m1 for *Pparδ*, Mm01191972_m1 for *Npc1l1*, Mm00436419_m1 for *Fxr*, Mm00488258_m1 for *Asbt*, Mm00434316_m1 for *Ibabp*, Mm00496899_m1 for *Mrp2*, Mm00551550_m1 for *Mrp3*, Mm01226381_m1 for *Mrp4*, Mm00521530_m1 for *Ostα*, Mm01175040_m1 for *Ostβ*, Mm01344139_m1 for *Pxr*, Mm00484152_m1 for *Cyp7a1*, Mm00470430_m1 for *Cyp27a1*, Mm00441421_m1 for *Ntcp*, Mm00445168_m1 for *Bsep,* Mm00433278_m1 for *Fgf15*, and Mm00501637_s1 for *Cyp8b1*. *Gapdh* (Mm99999915_g1) was used as the reference gene. Stress did not have an effect on the Ct values of *Gapdh* in the liver or small intestine; therefore it is applicable as a housekeeping gene control. Reactions were run on a CFX96TM Real-Time System (Bio-Rad, Hercules, CA) according to the manufacturer's instructions. The relative mRNA expression levels were calculated by the ΔΔ*C*_t_ method.

### Western blotting

Frozen livers and small intestinal sections were homogenized by sonication in modified radioimmunoprecipitation assay buffer (50 mmol/L Tris, 150 mmol/L NaCl, 0.1% SDS, 1% Triton X, 0.5% Na-deoxycholate) with protease inhibitor cocktail (Sigma-Aldrich). From the liver and small intestinal lysates, 50–70 *μ*g of protein per sample was fractionated with SDS-PAGE and transferred onto a nitrocellulose membrane by semidry blotting. After blocking in 5% (w/v) BSA, immunodetection was performed with the polyclonal rabbit SR-BI antibody (1:1000, Novus Biologicals, Littleton, CO), polyclonal rabbit ASBT antibody (1:150, Abcam, Cambridge, UK), or with polyclonal rabbit CYP7A1 antibody (1:200, Santa Cruz, Dallas, TX). Bound primary antibody was detected by horseradish peroxidase-conjugated anti-rabbit IgGs (Dako, Glostrup, Denmark) and enhanced chemiluminescence (ELC plus, GE Health Care, Buckinghamshire, UK). After stripping of the membranes with glycine stripping buffer (pH 2.5), *β*-actin was immunoblotted as a loading control (mouse anti- *β*-actin, 1:1500, Abcam). Optical densities of protein bands were quantified with ImageJ software (National Institute of Health, Bethesda, MD) and normalized to densities of corresponding *β*-actin bands.

### Analysis of fecal bile acids and neutral sterols by gas–liquid chromatography

Feces were collected over 24 h from the cages of individually housed mice on day 4 of the 5-day stress regime. Samples were dried for 24 h at 50°C and pulverized. Total bile acids and neutral sterols were extracted and analyzed by a gas–liquid chromatography (Grundy et al. 1965; Miettinen 1982) in a system (Agilent 6890N Network GC System, Agilent Technologies, Santa Clara, CA) equipped with a 50 m long nonpolar Ultra 1 capillary column for bile acids and Ultra 2 capillary column for neutral sterols. Standards (Sigma-Aldric and Steraloids Ltd, Newport, RI) were run to identify the following individual bile acids: cholic acid, chenodeoxycholic acid, *β*-muricholic acid, deoxycholic acid, lithocholic acid, isolithocholic acid, and epideoxycholic acid. Fecal neutral sterols included cholesterol, coprostanol, and the following plant sterols: campesterol, campestanol, stigmasterol, sitosterol, sitostanol and avenesterol.

### Other analyses

Serum total and HDL cholesterol and triglycerides as well as biliary cholesterol and phospholipids were measured by commercial kits (Roche, Basel, Switzerland and Cayman chemicals, Ann Arbor, MI). Serum corticosterone was quantified with a commercial ELISA assay (R&D Systems, Minneapolis, MN). Bile acids were extracted from dried feces in 75% (v/v) ethanol for 2 h at 50°C, and total bile acids in the fecal extracts and bile were measured with an enzymatic assay kit (Diazyme Laboratories, Poway, CA). Fecal cholesterol was quantified by the Amplex Red cholesterol assay kit (Thermo Fisher Scientific, Waltham, MA).

### Statistics

GraphPad Prism 5.0 software (La Jolla, CA) was used for all statistical analyses. When data were normally distributed, Student's unpaired *T*-test was used to compare differences between the control and stressed mouse groups. The nonparametric Mann–Whitney test was used for data that did not follow Gaussian distribution or whenever a small size of a dataset (*n* ≤ 7) did not allow the testing of normality. *P* ≤ 0.05 were considered statistically significant.

## Results

### Mice do not habituate to chronic intermittent stress in 2 weeks

Mice subjected to repeated restraint stress episodes for up to 14 days exhibited sharply elevated concentration of the stress hormone corticosterone after the final stress session suggesting a lack of habituation to the stressor at the level of the hypothalamo-pituitary-adrenocortical (HPA) axis (Fig.2A). Despite similar food consumption in the control and stressed mouse groups during the 5-day stress regime (Fig.2B), mice subjected to stress lost ~8% more of their body weight during the period (Fig.2C). Defecation increased during stress episodes (Fig.2D); however, the effect was transient and the total weight of the feces expelled during the final 48 h of the 5-day stress regime was similar in the control and stressed groups (Fig.2E).

### Chronic intermittent stress promotes macrophage-to-feces cholesterol transport by stimulating fecal excretion of bile acids but not that of cholesterol

**Figure 3:**
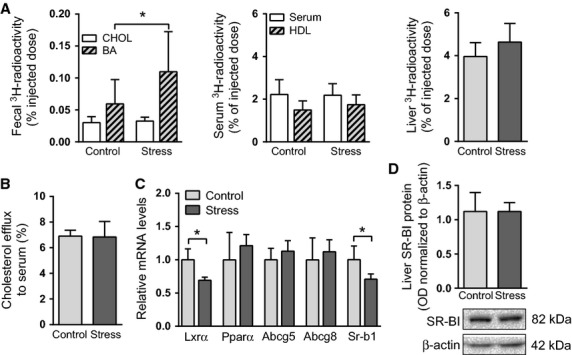
Chronic intermittent stress stimulates m-RCT without affecting cholesterol efflux or the expression of hepatic cholesterol transporters. (A) Mice were injected intraperitoneally with [^3^H]cholesterol-loaded macrophages. ^3^H-radioactivity (as % of injected dose) in fecal cholesterol (CHOL) and bile acid (BA) fractions, in total serum and HDL fraction, and in the liver was determined 24 h after the injection (*N* = 8–10 mice/group). (B) The capacity of serum (2.5% v/v in medium) to stimulate cholesterol efflux from J774A macrophage foam cells was measured in vitro (serum pools from 5 mice/group). (C) mRNA levels (relative units) of hepatic cholesterol transporters and transcription factors (*N* = 5–10 mice/group). (D) Hepatic scavenger receptor BI (SR-BI) protein level (as optical density, OD, normalized to that of *β*-actin) (*N* = 5 mice/group). *P < 0.05. All data are presented as mean + SD.

For the m-RCT in vivo assay, mice were injected intraperitoneally with [^3^H]cholesterol-loaded macrophages, and the transfer of labeled cholesterol from the macrophages to HDL, liver, and feces was measured within 24 h. When compared with control mice, animals exposed to repeated stress exhibited significantly increased fecal ^3^H-radioactivity, reflecting enhanced rate of m-RCT. Of note, the increase in the tracer levels in feces was fully due to a marked increase in the [^3^H]bile acid fraction (Fig.3A). Similar levels of serum and liver ^3^H-radioactivity in the stressed and control mice were observed. Neither did serum of the stressed mice, when compared with serum of the control mice, show any alteration in its capacity to accept cholesterol from [^3^H]cholesterol-loaded macrophage foam cells in vitro (Fig.3B). Evaluation of the expression of genes related to cholesterol transport in the liver of the stressed mice revealed that despite downregulation of the mRNA of liver X receptor *α* (LXR*α*), a key regulator of cholesterol homeostasis, the expression of its hepatic target genes *Abcg5* and *Abcg8,* encoding the canalicular cholesterol exporter ABCG5/8, was not altered in the stressed mice (Fig.3C). The mRNA levels of the scavenger receptor BI (SR-BI), a key hepatic cholesterol uptake receptor, were reduced in the stressed mice, but the SR-BI protein levels were similar to those of control mice (Fig.3D).

### Chronic intermittent stress impairs the intestinal absorption of bile acids but not that of cholesterol

**Figure 4:**
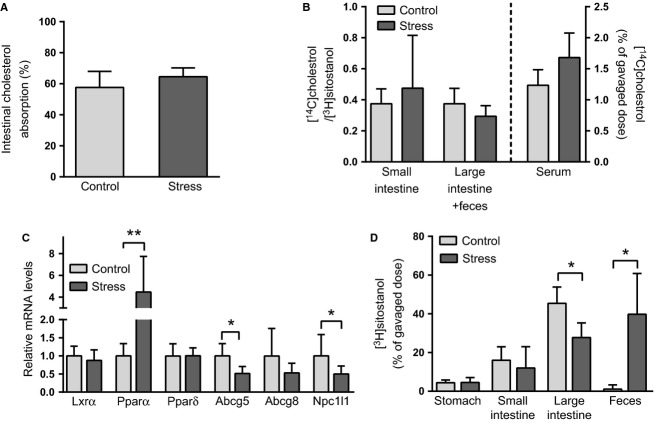
Chronic intermittent stress does not alter intestinal cholesterol absorption but it affects mRNA expression of intestinal cholesterol transporters and stimulates large intestinal transit. (A) Fractional cholesterol absorption efficiency (%) was measured by the fecal dual isotope method over 24 h (*N* = 6–10 mice/group). (B) Intestinal and fecal [^14^C]cholesterol/[^3^H]sitostanol ratios and the serum [^14^C]cholesterol content were measured 3 h after an oral gavage of [^14^C]cholesterol and [^3^H]sitostanol (*N* = 5 mice/group). (C) mRNA levels (relative units) of intestinal transcription factors and cholesterol transporters regulating cholesterol absorption (*N* = 5–15 mice/group). (D) [^3^H]sitostanol levels (as % of gavaged dose) in the gastrointestinal tract of control and repeatedly stressed mice 3 h after oral dosing (*N* = 10). *P < 0.05, **P < 0.01. All data are presented as mean + SD.

The fecal dual isotope method was applied to measure cholesterol absorption over the final 24 h of the 5-day stress regime. As shown in Figure4A, the percentage of cholesterol absorbed was similar in control and stressed mice. To detect also transient changes that might occur in cholesterol absorption during individual stress episodes, mice received by gastric gavage a mixture of [^14^C]cholesterol and [^3^H]sitostanol immediately before the final stress episode included in the 5-day stress regime, and the radioactivities in the contents of the small and large intestine and feces were measured after 3 h. Neither the [^14^C]/[^3^H] ratio in the intestine nor the [^14^C]cholesterol level in blood revealed significant changes in cholesterol absorption rate during the final stress episode (Fig.4B). When compared with controls, the stressed mice exhibited increased expression of *Pparα* and decreased expression of *Abcg5* and *Npc1l1* in the small intestine (Fig.4C).

**Figure 5:**
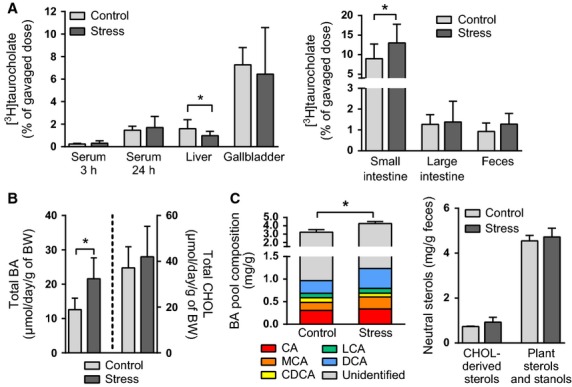
Chronic intermittent stress alters the intestinal distribution of [^3^H]taurocholate and stimulates the total fecal excretion of bile acids. (A) Distribution of orally gavaged [^3^H]taurocholate (as % of dose normalized to the recovery of an internal standard) in serum and liver, gallbladder, small and large intestine, and feces 24 h (or 3 h in the case of the first blood sample) after administration (*N* = 10 mice/group). (B) Total fecal bile acid (BA) and cholesterol (CHOL) excretion rate (*μ*mol per day per gram of body weight, BW) in repeatedly stressed and control mice during the final 48 h of the 5-day stress regime (*N* = 5 mice/group). (C) Composition of the bile acid pool (*N* = 5 mice/group) and neutral sterol pool (*N* = 3 mice/group) in feces excreted over 24 h on day 4 of the 5-day stress regime. CA = cholic acid, MCA = muricholic acid, CDCA = chenodeoxycholic acid, LCA = litocholic acid, DCA = deoxycholic acid. The cholesterol-derived neutral sterols included cholesterol and coprostanol, and the plant-derived neutral sterols and stanols included campesterol, campestanol, stigmasterol, sitosterol, sitostanol, and avenesterol. *P < 0.05. All data are presented as mean + SD.

A difference between control and stressed mice in the intestinal distribution of the nonabsorbable [^3^H]sitostanol (Fig.4D) indicated that transit through the large intestine was significantly increased by stress. Because bile acid absorption may be affected by variations in intestinal transit time, the efficiency of intestinal bile acid absorption was evaluated by measuring the distribution of orally gavaged [^3^H]-labeled taurocholate in mice subjected to intermittent chronic stress and in the respective control group. As shown in Figure5A, [^3^H]taurocholate levels decreased in the liver of the stressed mice, whereas no change was observed in serum or gallbladder. An increase in small intestinal [^3^H]taurocholate pointed toward reduced absorption in the stressed mice, although only a small fraction (~1%) of [^3^H]taurocholate was excreted in feces during 24 h, and this amount did not differ between control and stressed mice.

In contrast to taurocholic acid that is mainly absorbed by an active transporter-mediated mechanism in the ileum (Aldini et al. 1996), bacterial-produced secondary bile acids as well as unconjugated and uncharged bile acid species are absorbed passively in the distal intestine. To assess the effect of chronic intermittent stress on total fecal excretion of endogenous bile acids and on the composition of the fecal bile acid pool, total bile acids were measured enzymatically, and different bile acid species were separated by gas–liquid chromatography. When compared with control mice, the stressed mice exhibited increased fecal excretion of bile acids, but not of cholesterol, during the two final days of the 5-day stress regime (Fig.5B), a result that is well in line with the m-RCT data (Fig.3A). Gas–liquid chromatography analysis of feces produced on the 4th day of the 5-day stress regime confirmed the increase in total bile acid content, but did not reveal drastic changes in the composition of the fecal bile acid pool (Fig.5C). Also the composition of the fecal neutral sterol pool was similar in control and stressed mice.

**Figure 6:**
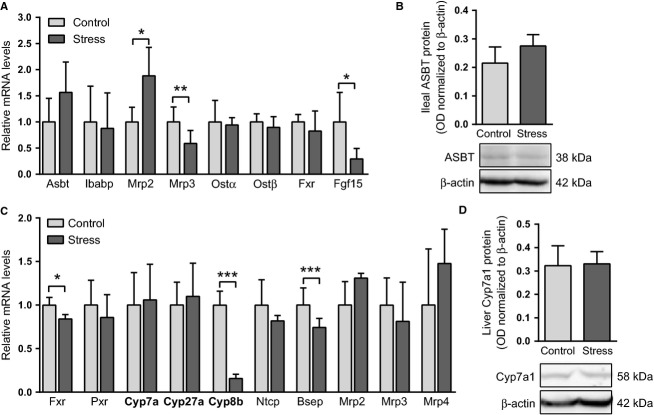
Effect of chronic intermittent stress on the intestinal and hepatic expression of transcription factors, enzymes, and transporters that govern the enterohepatic circulation of bile acids. (A) mRNA levels (relative units) of key bile acid transporters and the transcription factor FXR in the small intestine (*N* = 5–10 mice/group). (B) Ileal ASBT protein (as optical density, OD, normalized to that of *β*-actin) (*N* = 5 mice/group). (C) Hepatic mRNA levels (relative units) of the key bile acid transporters, enzymes of synthesis pathways, and regulating transcription factors (*N* = 5–15 mice/group). (D) Hepatic CYP7A1 protein expression (as optical density, OD, normalized to that of *β*-actin) (*N* = 5 mice/group). *P < 0.05, **P < 0.01, ***P < 0.001. All data are presented as mean + SD.

Analysis of intestinal mRNA expression revealed that the transcription of the apical sodium-dependent bile acid transporter (ASBT), the intracellular ileal bile acid-binding protein (IBABP), or the basolateral organic solute transporter *α*/*β* (OST*α*/*β*), all mediating the active absorption of major bile acid species, was not affected by stress (Fig.6A). Also ASBT protein levels were similar in stressed and control mice (Fig.6B). However, the mRNA of the multidrug resistance protein 2 (MRP2), an apical transporter that facilitates the efflux of sulphated and glucuronidated bile acids back into the intestinal lumen, was upregulated and that of the MRP3, a basolateral bile acid transporter alternative to OST*α*/*β*, was downregulated in the stressed mice when compared with controls. This suggests that alternative routes of bile acid absorption might have been affected by stress. Although the intestinal mRNA level of the intracellular bile acid sensor FXR was not changed, its target gene fibroblast growth factor 15 (FGF15) was downregulated in the stressed mice (Fig.6A).

Altogether, the data presented above show that the m-RCT stimulation observed in repeatedly stressed mice was derived from globally increased fecal bile acid excretion. Expression of ASBT, the key protein in the active bile acid absorption process, remained unchanged in the stressed mice. Nonetheless, the data suggest that passive and other alternative routes of bile acid absorption may have been hampered by chronic intermittent stress and the transiently increased large intestinal transit associated with it.

### The expression of CYP7A1 is not altered immediately after stress or during ensuing sedentary periods

**Figure 7:**
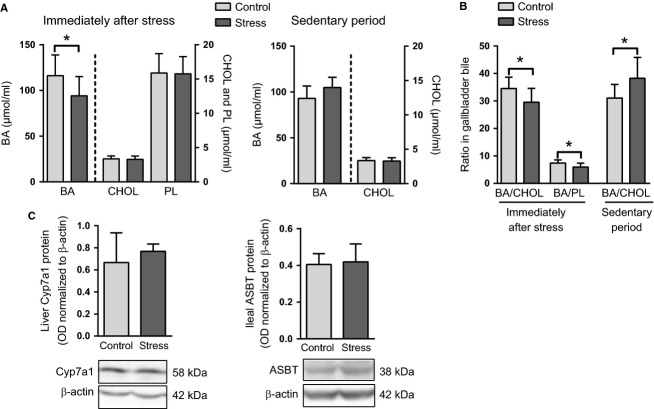
Gallbladder bile acid levels and protein expression of CYP7A1 and ASBT in repeatedly stressed and control mice immediately after stress and during a 4-h sedentary period. Gallbladder bile was collected immediately (day 5) or 4 h after stress (sedentary period, day 4) from control and stressed mice. All mice were fasted for 2 h before sample collection. (A) Bile acid (BA), cholesterol (CHOL), and phospholipid (PL) concentrations and (B) the bile acid/cholesterol (BA/CHOL) and bile acid/phospholipid (BA/PL) ratios were measured in the collected bile (*N* = 4–8 mice/group). (C) Hepatic CYP7A1 and ileal ASBT protein expression (as optical density, OD, normalized to that of *β*-actin) in control and repeatedly stressed mice after a 4-h sedentary period (*N* = 5 mice/group). *P < 0.05. All data are presented as mean + SD.

Gene expression of hepatic enzymes and transporters mediating bile acid synthesis and biliary flow was measured immediately after the final stress episode of the 5-day stress regime. Hepatic *Fxr*, the key nuclear factor orchestrating the regulation of bile acid synthesis and transport, was downregulated in the stressed mice when compared with controls (Fig.6C). The mRNA expression of cholesterol 7*α*-hydroxylase (CYP7A1) and sterol 27-hydroxylase (CYP27A1), encoding the first enzymes of the neutral and acidic bile acid synthesis pathways, respectively, was not altered whereas that of sterol-12*α*-hydroxylase (CYP8B1) was strongly downregulated in the stressed mice (Fig.6C). Control and stressed mice exhibited similar levels of CYP7A1 protein in the liver (Fig.6D). The downregulation of *Fxr* and its target gene *Bsep* in the liver of the stressed mice was associated with partial depletion of bile acids in the gallbladder bile immediately after stress (Fig.7A). This was reflected also by reductions in the bile acid/cholesterol and bile acid/phospholipid ratios (Fig.7B).

To assess the persistence of the stress-associated alterations and to identify potential compensatory changes occurring during the sedentary periods between every two consecutive stress sessions, a group of mice was euthanized 4 h after the first stress episode of day 4 of the chronic intermittent stress regime, and samples were collected from multiple sites for analysis. As shown in Figure7B, the stress-induced reduction in gallbladder bile acid concentration was not permanent; the bile acid/cholesterol ratio in the gallbladder bile was actually increased in the stressed group after a 4-h sedentary period when compared with the respective control group. The volume of bile obtained from the gallbladder (7.5 ± 2.5 *μ*L in the two control groups vs. 10.1 ± 3.8 *μ*L immediately and 8.6 ± 2.1 *μ*L 4 h after stress) did not significantly differ between groups. Western blotting of the hepatic CYP7A1 and the ileal ASBT revealed no differences in protein levels between the stressed and control mice after a 4-h sedentary period (Fig.7C). Serum bile acid and cholesterol levels measured immediately after stress and 4 h after stress (sedentary period) are listed in the Table1. Compared to controls, the stressed mice exhibited reduced concentrations of serum bile acids and cholesterol when measured immediately after stress. The levels of circulating cholesterol and bile acids did not differ between control and stressed mice when measured 4 h after stress (Table1).

Altogether, the data presented above show that stress transiently reduced serum and gallbladder bile acid levels and the serum concentration of non-HDL cholesterol. The reduced serum and gallbladder bile acid levels concur with the increased fecal bile acid loss (Fig.5B). Lack of ASBT or CYP7A1 upregulation in the stressed mice immediately and 4 h after stress suggests that the observed bile acid depletion did not induce compensatory increases in CYP7A1-mediated bile acid synthesis or in active ASBT-mediated bile acid absorption during daytime sedentary periods.

## Discussion

The stress response involves an array of mediators to adjust energy homeostasis according to need. During a hypermetabolic response evoked by repeated stress, hepatic gluconeogenesis is fueled by mobilized fatty acids, glycerol, and amino acids, resulting in a hyperglycemic state (Depke et al. 2008). The key stress hormones, catecholamines and glucocorticoids, and glucagon stimulate protein breakdown, whereas the stress-activated hormone-sensitive lipase degrades triglycerides in the adipose tissue (Depke et al. 2008; Konstandi et al. 2013). This results in loss of body weight which was evident also in our study after 5 days of chronic intermittent stress (Fig.2C). Increased circulating glucose and intracerebral lactate levels associated with the stress-induced hypermetabolic response may hamper VLDL production in the liver (Lam et al. 2007). This might be an underlying reason to reduced serum levels of total and non-HDL cholesterol observed by us (Table1) and others (Depke et al. 2008).

Also bile acids and their sensor FXR are involved in controlling the metabolism of glucose and lipids in the liver and intestine as well as energy expenditure in peripheral tissues (Watanabe et al. 2006; Lefebvre et al. 2009). In this light, complex interaction between glucocorticoids and bile acid homeostasis are conceivable. In our model of chronic intermittent stress, mRNA expression of the hepatic bile acid sensor FXR was modestly reduced immediately after stress exposure (Fig.6C). Furthermore, the transient decline in the level of serum bile acids (Table1), which are natural FXR-activating ligands, likely resulted in diminished FXR activity causing the observed downregulation of its hepatic target gene *Bsep* and its intestinal target gene *Fgf15* in the stressed mice (Fig.6A and C). Accordingly, bile acid concentration in the gallbladder bile was reduced immediately after stress exposure (Fig.7A). Because this reduction was no more detectable at 4 h post stress (Fig.7B), changes in FXR and BSEP likely occurred at the functional rather than transcriptional level. This conclusion is supported by the fact that alterations in the expression levels of BSEP seldom correlate with observed changes in hepatobiliary bile acid fluxes (Wolters et al. 2002; Out et al. 2014). Regulation of FXR activity in stress extends beyond changes in ligand availability: comprehensive studies by Lu and coworkers (Lu et al. 2012) have revealed that dexamethasone-activated glucocorticoid receptor (GR) is able to suppress the transactivation function of FXR by recruiting a corepressor, C-terminal-binding protein, to the complex formed by GR and FXR on the promoter of the small heterodimer partner (SHP), an important downstream effector of FXR.

In a steady state, the whole-body bile acid pool size remains constant because any depletion of bile acids will trigger hepatic de novo bile acid synthesis via suppression of FXR; that is, impaired ability of the hepatic FXR to activate SHP will allow another transcription factor, the liver receptor homologue 1 (LRH-1), to stimulate bile acid synthesis via CYP7A1 (Goodwin et al. 2000; Sinal et al. 2000; Rose et al. 2011). Reduced activity of the intestinal FXR will also relieve FGF15-mediated inhibition of the hepatic *Cyp7a1* (Inagaki et al. 2005). Despite downregulation of the hepatic *Fxr* and the intestinal *Fgf15*, CYP7A1 mRNA and protein expression were not affected by chronic intermittent stress (Fig.6). Moreover, the mRNA of CYP8B1, an enzyme that is responsible for the synthesis of the primary bile acid cholate along the CYP7A1-initiated pathway (Vlahcevic et al. 1997; Li-Hawkins et al. 2000; Schwarz et al. 2001), was even downregulated (Fig.6C). These results strongly suggest that bile acid synthesis was not affected by the present model of chronic stress.

Dexamethasone-treated mice typically exhibit induction of CYP7A1 (Sinal et al. 2000; Lu et al. 2012). Two mouse studies applying very similar stress models to ours reported increased transcription of *Cyp8b1* but not of *Cyp7a1* after 4-day exposures to repeated stress (Depke et al. 2008; Konstandi et al. 2013). Differences in the observations may arise from the elaborate FXR-dependent and -independent mechanisms that participate in the regulation of *Cyp7a1*; pathways relevant in the stress condition include those mediated by PPAR*α* and LXR*α*. LXR*α*, induced by buildup of cholesterol in the liver, stimulates *Cyp7a1* transcription in mice (Peet et al. 1998; Tobin et al. 2000; Stulnig et al. 2002). Repeatedly stressed mice exhibited reduced transcription of *Lxrα* (Fig.3C), a finding that is supported by studies showing counteractive roles for the GR and LXR*α* in stress (Stulnig et al. 2002; Steffensen et al. 2003). In contrast, PPAR*α*, a key stimulator of fatty acid uptake and oxidation in the liver and intestine, is activated during stress via circulating glucocorticoids and fatty acids (Lemberger et al. 1996; Bernal-Mizrachi et al. 2003; Konstandi et al. 2013). Treatment of mice with synthetic agonists of PPAR*α* results in inhibition of CYP7A1 and CYP27A1 in the liver (Hunt et al. 2000; Post et al. 2001). Stimulation of the hepatic PPAR*α*, although not evident at the mRNA level (Fig.3C), and the inhibition of *Lxrα* transcription might have prevented *Cyp7a1* upregulation and the consequent induction of bile acid synthesis after stress exposure in our study.

Changes in PPAR*α*, FXR and glucocorticoid activity do not only affect *Cyp7a1*, but also regulate the expression of the main transporters mediating the enterohepatic circulation of bile acids, namely ASBT, BSEP*,* and the hepatic Na^+^-taurocholate cotransporting polypeptide (NTCP) (Kok et al. 2003; Eloranta et al. 2006; Bunger et al. 2007; Rose et al. 2011; Lu et al. 2012). In a recent study by Out et al. (2014), male BALB-c mice were implanted with prednisolone-releasing pellets for 7 days which resulted in marked stimulation of bile acid absorption and biliary flow rates along with stimulation of *Asbt* transcription. The increased turnover and transhepatic flux of bile acids elevated circulating bile acid levels, diminished bile acid synthesis rate, and increased m-RCT (Out et al. 2014). This depiction differs markedly from our observations recorded in mice exposed to chronic intermittent stress. A key to the discrepancy may lie not only in the different abilities of glucocorticoids to affect bile acid transporters and regulator molecules (Rosales et al. 2013) but also in the distinct effects of stress mediators and exogenous glucocorticoids on gastrointestinal function (Saunders et al. 2002). Colonic motor responses to stress occur also in humans (Rao et al. 1998) and can be explained by the stress-induced bursts of corticotropin releasing hormone (CRH) that promptly increase colonic motility through stimulation of enteric motor neurons (Lenz et al. 1988). In contrast, administration of exogenous glucocorticoids typically suppresses the production and secretion of CRH in the neurons of the paraventricular nuclei of the hypothalamus (Plotsky et al. 1986; Girotti et al. 2007) which leaves peristalsis of the distal ileum and large intestine unaffected.

In a steady state, approximately 5% of the secreted biliary bile acids escape the small intestinal reabsorption phase during each enterohepatic cycle. Considering the number of cycles per day (~ 5–7 in mice), the amount of bile acids that enter the colon is not negligible. The escaped bile acids are metabolized by colonic bacteria, yielding more than ten different species of secondary bile acids in mice (Schwarz et al. 1996; Ridlon et al. 2006). The absorption of these secondary bile acids, and that of all unconjugated and uncharged bile acid species, is passive and thus depends on the charge and conjugation pattern of the bile acid species, and most importantly, on their transit rate through the colon (Schiff et al. 1972; Dowling et al. 1997; Veysey et al. 2001). The functional and transcriptional data collected from repeatedly stressed mice (Figs.2D, 5, 6) suggest that transiently increased colonic transit, occurring during stress exposure, compromised the passive absorption of bile acids in the large intestine. Importantly, habituation did not occur, and the unaltered cumulative stool production over the final 48 h of the stress regime (Fig.2E) can be explained by reduced poststress defecation which reflects the time required for refilling of the large intestine after its efficient emptying during the stress period.

Also human patients with Crohn's disease or diarrhea-prone irritable bowel syndrome (IBS) exhibit simultaneous alterations in intestinal transit and bile acid homeostasis: when compared with healthy subjects and IBS patients with constipation, these patients exhibit decreased intestinal transit time, reduced synthesis of secondary bile acids, increased total secretion of bile acids in the feces and, probably as a compensatory reaction, stimulated hepatic bile acid production (Kruis et al. 1986; Wong et al. 2012). In general, decreased production and absorption of secondary bile acids in the colon will yield a more hydrophilic bile acid pool that is less toxic and less efficient in downregulating bile acid synthesis (Heuman et al. 1989; Bayerdorffer et al. 1993). This may affect systemic bile acid homeostasis.

Several adaptive mechanisms aimed at maintaining homeostasis are activated during intermittent stress, some of them being apparently redundant or even situationally inappropriate. For instance, decreased NPC1L1-mediated import of cholesterol into enterocytes might have triggered downregulation of the intestinal *Abcg5/8* (Fig.4C) via LXR*α*, the transcription factor which is activated by cholesterol derivatives and positively regulates *Abcg5/8* and *Abca1* (Larrede et al. 2009; Engelking et al. 2012). However, as mere downregulation of the sterol efflux pump ABCG5/8 is likely to only minimally enhance the efficiency of intestinal cholesterol absorption (Lee-Rueckert et al. 2013), further adaptive mechanisms probably contributed to the restoration of cholesterol absorption in the repeatedly stressed mice which exhibited *Npc1l1* downregulation at the mRNA level (Fig.4C). Although the intestinal PPAR*α*-initiated regulatory pathway that resulted in functionally altered cholesterol absorption in acutely stressed mice (Silvennoinen et al. 2012) was interrupted in the repeatedly stressed mice, PPAR*α* remains a potential regulator of intestinal bile acid homeostasis in repeatedly stressed mice due to its effects on FXR-FGF15 signaling (Zhou et al. 2014) and intestinal motility (De Vogel-van den Bosch et al. 2008).

In summary, the aim of this study was to shed light on the complex relationship between psychological stress and atherosclerosis (Rozanski et al. 1999) by investigating the effects of chronic intermittent stress on the fluxes of cholesterol and bile acids along the m-RCT pathway. Mice exposed to repeated restraint stress episodes exhibited enlarged fecal pool of bile acids derived from macrophage-cholesterol. Globally increased fecal excretion of bile acids, decreased serum and gallbladder bile acid levels, and reduced transcription of hepatic *Fxr* and its target genes *Bsep* and *Fgf15*, all agree with the notion that intestinal bile acid absorption in the stressed mice was decreased. As the expression of ASBT was not altered, the transient increases in intestinal transit associated with repeated stress exposure likely hampered passive and alternative routes of intestinal bile acid absorption. Interestingly, the apparent bile acid depletion did not induce an immediate compensatory increase in CYP7A1-mediated bile acid synthesis.

As elegantly discussed by Dhabhar (2009), the evolutionary ancient stress reaction, as one of the nature's fundamental defense and survival mechanisms, harbors a wide array of mediators and targets, so creating multiple complex networks and pathways. Within these networks, multiple regulatory responses not necessarily representing the evolutionary early stages of a survival game have evolved. Such systems may have evolved as safety margins for survival in various stress situations, timing being an example of a key determinant which influences the direction (enhancing vs. suppressive) of the effects of stress. Against this background, we may surmise that the findings of this study establish a new potentially antiatherogenic mechanism for chronic intermittent psychological stress. Yet, atherosclerosis is not a disease of mice, which are “HDL animals” (Capman 1980). Accordingly, no atherosclerosis-related evolutionary advantages of stress-dependent beneficial changes in m-RCT can be effective in these animals. Moreover, as atherosclerosis in man is an age-related disease of modern westernized humans which manifests itself beyond the reproductive period (Wick et al. 2003; O'Keefe et al. 2004), it is not likely that even in humans there have been any direct evolutionary advantages due to beneficial (if any) effects of chronic stress on body cholesterol homeostasis.

In summary, the present data highlight the fact that changes in cholesterol and bile acid homeostasis induced by chronic stress cannot be directly extrapolated from data obtained when applying acute stress, or when treating mice with exogenous glucocorticoids. More studies investigating the physiological stress responses, especially in chronic experimental settings in which habituating mechanism become central, are needed to uncover the role of psychological stress in the regulation of m-RCT and ultimately, in atherosclerosis susceptibility of man.
